# Electrophysiological experiments in microgravity: lessons learned and future challenges

**DOI:** 10.1038/s41526-018-0042-3

**Published:** 2018-03-29

**Authors:** Simon L. Wuest, Benjamin Gantenbein, Fabian Ille, Marcel Egli

**Affiliations:** 1 School of Engineering and Architecture, Institute of Medical Engineering, Space Biology Group, Lucerne University of Applied Sciences and Arts, Hergiswil, Switzerland; 2 Institute for Surgical Technology and Biomechanics, Tissue and Organ Mechanobiology, University of Bern, Bern, Switzerland

## Abstract

Advances in electrophysiological experiments have led to the discovery of mechanosensitive ion channels (MSCs) and the identification of the physiological function of specific MSCs. They are believed to play important roles in mechanosensitive pathways by allowing for cells to sense their mechanical environment. However, the physiological function of many MSCs has not been conclusively identified. Therefore, experiments have been developed that expose cells to various mechanical loads, such as shear flow, membrane indentation, osmotic challenges and hydrostatic pressure. In line with these experiments, mechanical unloading, as experienced in microgravity, represents an interesting alternative condition, since exposure to microgravity leads to a series of physiological adaption processes. As outlined in this review, electrophysiological experiments performed in microgravity have shown an influence of gravity on biological functions depending on ion channels at all hierarchical levels, from the cellular level to organs. In this context, calcium signaling represents an interesting cellular pathway, as it involves the direct action of calcium-permeable ion channels, and specific gravitatic cells have linked graviperception to this pathway. Multiple key proteins in the graviperception pathways have been identified. However, measurements on vertebrae cells have revealed controversial results. In conclusion, electrophysiological experiments in microgravity have shown that ion-channel-dependent physiological processes are altered in mechanically unloaded conditions. Future experiments may provide a better understanding of the underlying mechanisms.

## Introduction

Living organisms strongly adapt to their daily mechanical load, and cells have shown responses to various mechanical stimuli in in-vitro experiments.^1–7^ However, it is still not fully understood how cells transform mechanical stimuli into biological responses. Among other mechanisms, mechanosensitive ion channels (MSCs) are thought to be key players (reviewed in refs. ^3,5,7–10^). Advances in electrophysiological experiments have shown that multiple mechanically induced physiological responses involve the activation of specific ion channels. However, the physiological function is not clear for many identified MSCs.^11–13^ Vice versa, multiple physiological functions are thought to rely on MSCs, but the pore-forming protein could not be identified.^14^ Various in-situ electrophysiological experiments have been developed to elucidate these unknowns. In this context, microgravity platforms have revealed interesting results: They allow for the study of biological processes in a mechanically unloaded condition. Furthermore, exposure to microgravity leads to a series of physiological adaption processes (reviewed in refs.^15–17^).

This review article outlines electrophysiological experiments performed in microgravity. After a general introduction to the mechanosensitivity of ion channels, various electrophysiological experiments conducted on multiple microgravity platforms are summarized. These experiments have shown an influence of gravity on all hierarchical levels of organization, from the cellular level to the organ. Furthermore, the gravitational effects on calcium signaling are discussed. The findings indicate that, despite the technical challenges, such experiments help to better understand how mechanical forces affects electrophysiological mechanisms.

## Mechanosensitivity of ion channels

Patch clamping electrically isolates an area of the cell’s membrane and enables electrophysiological recordings with high special resolution.^18^ Many MSCs have been discovered by aspiring a membrane patch with a glass micro-pipette and stretching it by suction (reviewed in ref. ^19^). MSCs (also referred as stretch activated channels) are characterized by their conformational change in response to mechanical load and the resulting transition into an open or closed state (reviewed in ref.^20^). MSCs are believed to play important roles in mechanosensitive pathways, allowing for cells to sense their mechanical environment (reviewed in refs. ^3,5,7–10^).

Along with the increasing numbers of identified mechanosensitive ion channels,^11,21,22^ the elucidation of the physiological function of specific MSCs has also progressed. For instance, the bacterial large conductance mechanosensitive channel (MscL) and small conductance mechanosensitive channel (MscS) are activated by membrane tension just below rupture tension of the lipid bilayer. Thereby, they function as “pressure relieve valves” and protect the cell from lysis in case of extreme osmotic swelling, such as after rainfall.^1,23–27^ MEC-4 (a member of the DEG/ENaC family) was identified as a mechanotransducer in *Caenorhabditis elegans*. External force activates mechanoreceptor currents in the touch receptor neurons.^28^ Similar, MSCs in the dorsal root ganglia of sensory neurons in vertebrates are activated by mechanical stimuli, thereby converting the mechanical stimuli into an electrical signal. Piezo2 and TRPA1 (transient receptor potential cation channel, first member in the ankyrin subfamily) are among the potential MSC candidates (reviewed in ref. ^29^). Additionally, in the inner ear of mammals, mechanically sensitive hair bundles protruding apically from hair cells transduce auditory and vestibular stimuli. Bundle deflection caused by sound-induced vibrations (auditory) or movement of the overlying otolithic membrane (vestibular) directly opens cation-permeable MSCs in the hair cell (reviewed in ref. ^14^). However, the pore-forming protein could not be fully identified.^30^

Currently, four eukaryotic channel families are thought to contain mechanosensitive members: the degenerin/epithelial sodium channels (DEG/ENaC), transient receptor potential channels (TRP), two-pore-domain potassium channels (K_2P_) and MscS-like channels (reviewed in refs.^10,11^). “MSCs are extremely diverse at a molecular level”^19^ and no “force-sensing domain” could be identified yet.^21^ For many MSCs it is unclear how the channel is coupled with the mechanical force, which is the subject of ongoing research. Some specific channels are thought to be linked either directly or indirectly to the cytoskeleton or the extracellular matrix. Other channels are believed to interact only with the surrounding lipids (reviewed in refs.^19–21,31,32^). For the latter channels, the channel-gating mechanism could be determined by the membrane properties, lipid mismatches and far-field tension (reviewed in ref.^19–21^). The properties of the lipid bilayer are known to respond to various changing conditions such as temperature,^33^ deformation,^34^ pH,^35^ specific ions,^36^ and gravity.^37^

The physiological function of many ion channels that have been identified to be mechanosensitive is still unknown.^11^ To add further complexity, various MSC channels can be activated through multiple pathways (reviewed in^22^). TRPC1 (transient receptor potential channel 1), for instance, is thought to be activated by the depletion of intracellular calcium-stores (store-operated calcium influx), through interactions with inositol 1,4,5-trisphosphate receptors (IP_3_Rs) or mechanically (stretch activated; reviewed in ref. ^38^). Likewise, TRPV4 (transient receptor potential cation channel, the fourth member in the vanilloid subfamily) is activated in response to hypotonic environments, membrane stress and moderate heat (24–38 °C; reviewed in ref. ^39^). One reason why the physiological function of many MSCs is unknown is because their mechanosensitivity has been detected in patch-clamping experiments with highly stressed plasma membranes.^20^ However, the cell membrane is thought to be relaxed under normal physiological conditions.^40^ Therefore, some channels that are considered to be MSCs might not belong to a physiological force-sensing system at all. Yet, some channels that are not considered to be MSCs are also known to be sensitive to membrane stretch. The voltage-dependent K^+^ channel (Kv), for instance, shows sensitivity to small mechanical perturbations of the membrane.^41^ Therefore, sensitivity to membrane tension could be a much more general property than commonly thought.^21^

Various experiments have been developed to determine the physiological function of MSCs, which have exposed cells to various mechanical loads in situ. In such electrophysiological experiments, cells have been exposed to cell stretch,^42–45^ shear flow,^46–48^ membrane indentation,^49–51^ osmotic challenges,^52,53^ hydrostatic pressure^54–56^ and other loading conditions. In line with these experiments, mechanical unloading, as experienced in microgravity, represents an interesting condition, since exposure to microgravity leads to a series of physiological adaption processes (reviewed in refs. ^15–17^).

## Electrophysiological experiments in microgravity

### Effects at the cellular and tissue levels

**Fig. 1:**
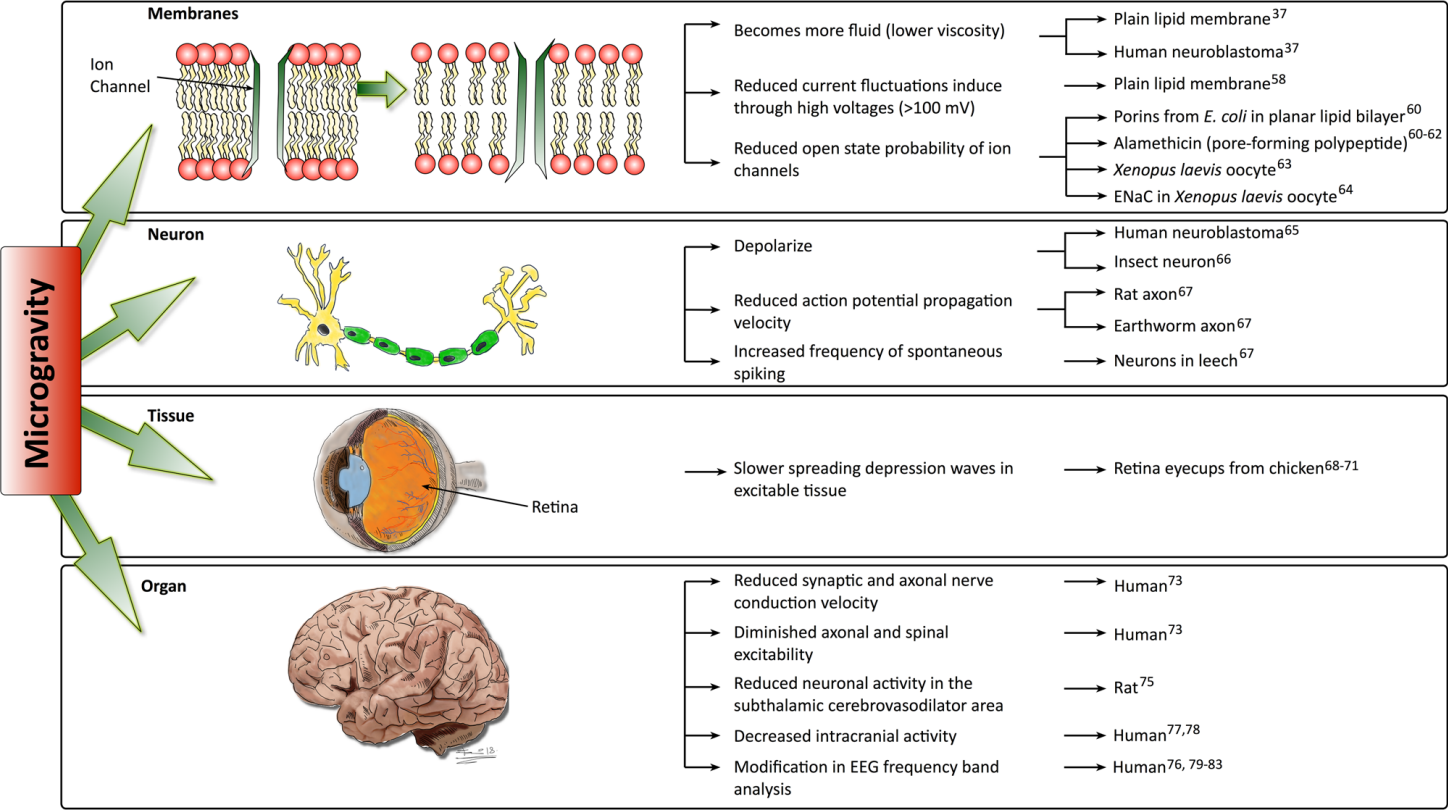
Effect of microgravity on cellular and organ functions that depend on ion channels. Gravity affects ion-channel-dependent physiological functions at all level of organization, from the membrane and the channels to the whole organism (left column). The middle column indicates microgravity induced effects and the right column indicate the specimen in which these effects have been observed. The images (left column) illustrate representative structures of microgravity exposed specimens

A number of electrophysiological experiments have been conducted in microgravity conditions. They have demonstrated that microgravity influences biological functions depending on electrophysiological properties at all levels of the hierarchical organization, from the membrane to the whole system (Fig. 1). Plain lipid membranes and membranes of the human neuroblastoma cell line SH-SY5Y become more fluid (lower viscosity) with decreasing gravity during parabolic flights.^37^ By applying high electrostatic potentials across plain lipid membranes (>100 mV), current fluctuations can be induced (reviewed in refs.^57^). This membrane conductance was reduced at lower gravity levels (during parabolic flight). The authors speculated that the increased membrane fluidity in microgravity could accelerate the repair of membrane structure defects.^58^ In the same experiment, the electrical capacity of plain lipid vesicles was slightly higher in hypergravity and microgravity. However, the authors could not rule out that the effect was due to changes in membrane geometry.^58^

As discussed above, the properties of lipid membranes could directly affect the kinetics of ion channels (see section “Mechanosensitivity of ion channels”). One could therefore expect gravity to influence specific ion channels as well. An early experiment on liposome-reconstituted cardiac gap junctions did not show altered channeling activity during parabolic flights.^59^ Later experiments showed that the open-state probability of porins isolated from *Escherichia coli* and incorporated into planar lipid bilayers decreased under microgravity and increased under hypergravity.^60^ Also, alamethicin, an artificial pore-forming polypeptide, shows reduced activity under microgravity and hypergravity.^60–62^ Furthermore, native oocytes from *Xenopus laevis*, as well as oocytes that overexpress epithelial sodium channels (ENaC), demonstrate reduced membrane conductivity under microgravity and increased conductivity under hypergravity (in parabolic flights).^63,64^ Ion channels are the key mediators of electric resting and action potentials (APs) in excitable cells. Neuronal cells (from the neuroblastoma cell line SH-SY5Y) hyperpolarized under higher gravity and depolarized under microgravity (parabolic flights).^65^ Likewise, insect neurons (SF21) depolarized in microgravity (drop tower experiment).^66^ AP kinetics are gravity dependent as well. The AP propagation velocity appeared to decrease under microgravity conditions and increased under hypergravity conditions in intact earthworms and isolated axons of rats and earthworms.^67^ The frequency of spontaneously spiking neurons in leeches increased in microgravity during a drop tower experiment.^67^ In agreement, excitable tissue is affected by gravity as well. Spreading depression (SD) waves in neuronal tissue are depolarization waves that are followed by a refractory period. The velocity of SD waves of retinal eyecups from chickens was slower under microgravity and faster under hypergravity (parabolic flights and centrifuges).^68–71^ However, the SD waves were faster during a sounding rocket mission (TEXUS).^68^ The authors speculated that a possible adaptation effect from the launch could have been the reason for this disagreement.

### Effects at the organ and system levels

Microgravity-induced effects have also been observed at the organ and systemic levels. Stimulating the posterior tibial nerve leads to involuntary and nearly instantaneous muscle contractions (neuromuscular reaction) of the soleus muscle.^72,73^ Experiments during parabolic flights “indicated that synaptic and axonal nerve conduction velocity, as well as axonal and spinal excitability are diminished with reduced gravitational forces […] and increased […] in hypergravity”.^73^ Kohn and Ritzmann reviewed the influence of gravity on neuromuscular systems in more detail.^74^

Multiple measurements on brain activity (electroencephalography, EEG) have been conducted under reduced-gravity conditions on animals and human subjects. Rats showed reduced neuronal activity in the subthalamic cerebrovasodilator area, a key area in controlling cerebral blood flow, in low-gravity conditions (parabolic flight).^75^ Recordings of the slow cortical potential (SCP) during parabolic flights showed that the SCP shifted in a positive direction for 4 out of 9 subjects during microgravity. A negative shift during microgravity was recorded for 3 of the subjects, and no significant reaction could be detected for 2 of the subjects. A positive shift of the DC potential indicates lower excitability of the central nervous system and inhibition of the cortical network.^76^ EEG recordings from a passenger in an aerobatic plane performing parabolic maneuvers showed decreased intracranial activity during the microgravity phase with both open and closed eyes. Statistically significant differences could be detected in the left occipital lobe and the right temporal lobe.^77,78^

Frequency band analyses of EEGs recorded in microgravity have revealed controversial results. The power of the spontaneous mu and alpha rhythms (8–12 Hz) recorded in the eyes-closed state increased in microgravity. The experiments were conducted over the course of three space flights.^79,80^ Beta-2 EEG activity (18-35 Hz) in the right superior frontal gyrus was inhibited in microgravity during parabolic flights.^81^ The same group also found an increase in brain activity inflight as compared to preflight. They interpreted this as a sign of increase in arousal due to the uncommon environment inflight.^82^ Another group also found a decrease in beta amplitude in microgravity on 9 subjects during parabolic flights, which indicated a lower arousal in microgravity.^76^ Marušič et al. reviewed the effects of various gravity levels on the brain (EEG recordings).^83^

Several astronauts have experienced heart rhythm disturbances during space flight missions. However, it was not clear if these arrhythmias were caused by microgravity or a stressful psychologically situation (reviewed in ref. ^84^).

## Gravitational effects on calcium signaling

### Introduction to calcium signaling

In the context of electrophysiological experiments performed in microgravity, calcium signaling represents an interesting pathway: (1) Calcium signaling involves the direct action of calcium-permeable ion channels. (2) Specific gravitactic cells have linked gravitropism to calcium signaling.

Calcium (Ca^2+^) is a ubiquitous intracellular signal responsible for controlling numerous cellular processes, including the cell cycle, proliferation, differentiation, apoptosis and cytoskeletal remodeling (reviewed in refs.^85,86^). The concentration of free Ca^2+^ in cytosol is tightly regulated by the combined action of channels, buffers, pumps and exchangers.^87^ At rest, the cytosolic Ca^2+^ concentration is around 100 nM. During Ca^2+^ signaling, the free Ca^2+^ in the cytosol transiently increases to roughly 1000 nM (reviewed in ref. ^86^). The principle Ca^2+^ sources during Ca^2+^ signaling are the extracellular environment and internal Ca^2+^ stores (primarily located in the endoplasmic or sarcoplasmic reticulum). Specific Ca^2+^-permeable ion channels (transiently) open and allow Ca^2+^ ions to rush into the cytosol, along the electrochemical gradient (reviewed in ref.^87^). From the inflowing Ca^2+^, only a very small quantity ends up as free Ca^2+^. Most of it is rapidly sequestered by Ca^2+^ buffers and effectors (reviewed in ref. ^87^). Generally, Ca^2+^ ions quickly encounter a binding protein and do not diffuse far (around 0.1–0.5 μm).^88,89^ Therefore, Ca^2+^ signaling can be extremely localized (reviewed in ref.^89^). Each cell type expresses a unique set of proteins to create a Ca^2+^ signaling system with different spatial and temporal properties (reviewed in refs. ^86,87^). Since several Ca^2+^-permeable ion channels have been shown to be sensitive to mechanical load, it is speculated that mechanical load could very well interfere with Ca^2+^ signaling.^85^

### Calcium signaling in gravitaxis

Graviperception is linked to Ca^2+^ signaling in specific gravitatic organisms, such as *Euglena* and *Arabidopsis thaliana*. The unicellular algae *Euglena* migrate vertically in the water column to an optimal position. Besides being phototactic, *Euglena* show an evidently negative gravitaxis (swimming against the gravity vector). *Euglena* are slightly denser than their surrounding water. The gravity-induced pressure of the whole cell body activates specific Ca^2+^-permeable MSCs at the front end of the cell.^90,91^ A transient receptor potential-like (TRP) channel has been identified as the presumable MSC.^92^ The entering Ca^2+^ binds to a specific calmodulin,^93^ which then activates cAMP-producing adenylyl cyclase.^94^ The cAMP subsequently activates a specific protein kinase A,^95^ which is thought to phosphorylate proteins inside the flagellum, resulting in a corrected swimming direction (reviewed in^96,97^). Accordingly, *Euglena gracilis* showed a transient Ca^2+^ signal when going from lower to higher accelerations during parabolic flights.^98,99^ During a sounding rocket flight (MAXUS 3) with an inflight centrifuge, *Euglena longa* showed an intermediate Ca^2+^ fluorescence signal in microgravity. The signal increased steeply with increasing acceleration by centrifugation.^100^ Also, the unicellular algae *Astasia* showed an increase in Ca^2+^ signal upon acceleration and a decrease in microgravity conditions during a sounding rocket experiment (MAXUS 3).^101^ Gravitaxis of *Euglena* has been reviewed in detail by Häder et al.^96,97^

Gravity sensing is thought to be mediated by Ca^2+^ in higher plants as well.^102–107^ However, the molecular mechanisms are not well understood.^103,108^ Gravity stimulation by parabolic flight induced a delayed increase in cytosolic Ca^2+^ in *A. thaliana* seedlings. Isolated *A. thaliana* cells showed an increase in intracellular Ca^2+^ concentration under low gravity and a decrease under hypergravity (parabolic flight).^109,110^

### Calcium signaling in cells of vertebrates

Recordings of the intracellular concentrations of free Ca^2+^ in animal cells under various gravitational loads have revealed controversial results so far. The intracellular Ca^2+^ concentration of neuronal cells decreased under microgravity during a drop tower experiment.^111^ In contrast, the intracellular Ca^2+^ concentration increased in a human neuroblastoma cell line (SH-SY5Y) under hypergravity and microgravity during a parabolic flight, which was believed to be due to a memory or hysteresis effect resulting from an increased Ca^2+^ concentration during the hypergravity phase.^112^ Still, recent data from the same human cell line (SH-SY5Y, undifferentiated state) showed that intracellular Ca^2+^ concentration increased under lower gravity and decreased under higher gravity (parabolic flight).^113^ Furthermore, findings by our group have shown that a microgravity-induced shift in free Ca^2+^ is cell-cycle dependent in mammalian cells (parabolic flight; publication in preparation). In a pilot study on native *Xenopus laevis* oocytes, a Ca^2+^-dependent current tended to be smaller under microgravity than under 1 g conditions (parabolic flight).^114^

## Methods used in electrophysiological microgravity experiments

Taken together, only limited numbers of electrophysiological experiments, especially at the cellular or subcellular level, have been conducted in microgravity conditions. In addition to limited access to microgravity platforms, classical electrophysiological techniques require delicate micromanipulation, which is not compatible with the high mechanical load generated on microgravity platforms. An attempt to use classical patch clamping in parabolic flights was discontinued because the aircraft’s vibrations frequently destroyed the patch and the recorded data had a poor signal-to-noise ratio.^66^ Even the mechanical disturbance caused by the release of the capsule in drop tower experiments was enough to destroy the patch. Only 3 out of 16 drops were successful during a previous drop tower campaign.^67^ An attempt to fly *Xenopus laevis* oocytes on a sounding rocket failed due to the high mechanical load during launch.^115^ Parabolic flight experiments with a two-electrode voltage clamp (TEVC) on *Xenopus laevis* oocytes have been discontinued due to practical manipulation difficulties. The oocytes were impaled with two micro-pipettes under a microscope in flight.^63^ Therefore, robust setups are required that need little micromanipulation. An adapted and non-invasive macro patch-clamp technique was used successfully on *Xenopus laevis* oocytes during parabolic flights.^64,114^ Also, the Port-a-Patch device from Nanion Technologies GmbH (Munich, Germany), employing a planar patch-clamp technique, was successfully used on a glioblastoma cell line (SNB19) during parabolic flights.^116^ Both setups require only simple in-flight manipulations and do not require a microscope. Voltage-sensitive or Ca^2+^-sensitive fluorescent dyes, which were recorded by optical means, have been successfully used multiple times.^98,101,111–113^ However, cytotoxicity, dye internalization and photo bleaching can limit the duration of the experiment.^117^ For instance, Meissner et al. reported degeneration of the cells while evacuating the drop tube in drop tower experiments.^111^

The extensive work identifying the molecular mechanisms responsible for gravitaxis in *Euglena* (see section “Calcium signaling in gravitaxis”), is a good example that microgravity experiments start on ground. By using pharmaceutical blockers and ionophores, it was shown that Ca^2+^ signaling is involved in gravitactic orientation (reviewed in ref. ^97^). Experiments employing the Ca^2+^-dependent fluorescent dye Calcium Crimson showed an increase in free Ca^2+^, during the reorientation of the cells along the gravity vector. Microscopic visualizations revealed a bright fluorescent signal at the front of the cell, indicating the location of the Ca^2+^-permeable channels.^90^ The responsible MSC could later be identified by selective gene expression knockdown, using RNA interference (RNAi).^92^ As described previously, multiple experiments on *Euglena*, and similar organisms, were conducted during parabolic flights and sounding rocket missions. For these experiments voltage-sensitive or Ca^2+^-sensitive fluorescent dyes were successfully employed.^98–101^ The work on entangling the molecular pathway responsible for gravitaxis in *Euglena* (reviewed in refs. ^96,97^) illustrates that we now have the tools to accomplish such challenges. Especially fluorescent probes in combination with an optical system proved to be useful tools on microgravity platforms.

## Conclusion and outlook

The results of the successful experiments suggest that gravity influences cellular and organ functions depending on ion channels. Generally speaking, it seems that the open-state probability of ion channels is lower and the kinematics is slower in low-gravity conditions. This could indicate that sensitivity to mechanical load could be a rather general property of ion channels. Since the channel-gating properties may be directly influenced by the membrane properties^19–21^, which are also gravity dependent,^37^ the membrane might be the primary structure of a cellular gravity-sensitive system. How gravity affects Ca^2+^ signaling in animal cells remains unclear. Since several Ca^2+^-permeable ion channels are also mechanosensitive^85^ and Ca^2+^ signaling is involved in the graviperception of specific plants, gravity might interfere with Ca^2+^ signaling in non-specialized cells as well.

Many of the performed experiments have been “black box” experiments that did not allow mechanosensitive molecular identities to be identified. Recent advances in engineering (e.g., automation, micro technology, and fast data acquisition) and molecular biology (e.g., pharmaceutical substances, reporter dyes, siRNA and genetic manipulation) will provide a promising toolkit for developing even more advanced experiments. In our opinion, future studies may be aimed at identifying the molecular mechanisms responsible for the rapid physiological adaptations seen in unloaded conditions.^15–17^ As compared to classical “rinse-and-fix” type studies,^118^ electrophysiological experiments allow living cells to be observed continuously and in real time. This is a major advantage when experimenting on microgravity platforms, since transitions to hyper-, micro- or normal gravity can be directly observed.

Experiments in the mechanically unloaded condition of microgravity have revealed important and sometimes surprising results. Even though gravity becomes a very small force at the cellular or subcellular level, multiple experiments have shown that microgravity greatly influences the function of isolated cells.^17,118–122^ However, to date it is unclear whether cells sense microgravity directly or indirectly. In microgravity, all gravity-dependent physical processes are altered and thus, sedimentation (and buoyancy), hydrostatic pressure difference and convection are (almost) absent in a static cell culture system. This changes the microenvironment of a living cell, which might influence its normal behavior.^123^ In previous reviews on gravitational cell biology, scientists argued that nonspecialized cells are unable to sense unit gravity. At the cellular level, gravitational forces are much smaller than other forces such as electrical forces, thermal noise and chemical energies.^123^ Therefore, the response of microgravity exposed cells, must be the result of an altered microenvironment. However, in the light of the before discussed results, this view may have to be challenged. Almost instantaneous responses to microgravity observed on membranes, ion channels and isolated cells are unlikely to be triggered by reduced sedimentation or convection.

As discussed in this review, the sensitivity of ion channels to mechanical load may not be limited to MSCs but could be a rather general property.^21^ In fact, mechanosensitivity might be a general property at the molecular level. For instance, many of the enzymes and substrates involved in DNA synthesis, RNA processing, protein synthesis and glycolysis are only functional when immobilized on insoluble scaffolds (reviewed in refs.^124,125^). Even the lifetime of non-covalent bonds decreases under force.^126^ Surprisingly, the molecular processes of microtubule self-organization in a cell-free system also appear to be gravity dependent.^127–129^ In conclusion, electrophysiological experiments in microgravity have shown that ion-channel-dependent physiological processes are altered in a mechanically unloaded condition. Future experiments shall be aimed at better understanding the underlying mechanisms.
